# Identification of G4-regulated immune-related drug targets for prostate cancer based on G4 screen and machine learning

**DOI:** 10.3389/fimmu.2026.1806289

**Published:** 2026-06-10

**Authors:** Liu Ge, Yue Hou, Xuan Huang, Jiawei Zhang, Lin Yao, Saisai Chen, Likai Mao, Wei Ning, Han Liang, Nini Shi, ChangBin Yang, Dongfang Jiang, Yuanji Shen, Bing Zheng, Chuanjun Shu, Minhao Zhang

**Affiliations:** 1 Department of Urology, Xishan People’s Hospital of Wuxi City, Wuxi, Jiangsu, China; 2 Department of Urology, Affiliated Zhongda Hospital of Southeast University, Nanjing, Jiangsu, China; 3 Military Medical Innovation Center, Fourth Military Medical University, Xi’an, China; 4 Reproductive Medical Center, Jinling Hospital Affiliated to Medical School of Nanjing University, Nanjing, Jiangsu, China; 5 Department of Oncology, Jinling Hospital Affiliated to Medical School of Nanjing University, Nanjing, Jiangsu, China; 6 The People’s Hospital of Danyang, Affiliated Danyang Hospital of Nantong University, Danyang, Jiangsu, China; 7 Department of Urology, Nanjing Pukou Hospital of Traditional Chinese Medicine (TCM), Nanjing, Jiangsu, China; 8 Department of Urology, Southeast University Affiliated Nantong First People’s Hospital, Nantong, Jiangsu, China; 9 Department of Bioinformatics, School of Biomedical Engineering and Informatics, Nanjing Medical University, Nanjing, China

**Keywords:** G4, IKBKB, immune-related drug targets, machine learning, prostate cancer

## Abstract

**Background:**

G-quadruplex (G4) structures are important epigenetic regulators and potential therapeutic targets in cancer. However, their role in prostate cancer, particularly in relation to the immune microenvironment, remains poorly understood.

**Methods:**

We performed BG4 ChIP-seq to map genome-wide G4 structures in the prostate cancer cell line C4-2. Bioinformatics analyses integrated G4-associated genes with immune pathway enrichment and machine learning algorithms (LASSO, SVM-RFE, GBM, Naïve Bayes, and GLM) to identify hub genes in prostate cancer progression. Clinical data from GTEx, TCGA, and HPA were analyzed for expression and survival. Functional validation included qPCR, CCK-8, colony formation, and wound-healing assays. Druggability was assessed using DrugnomeAI, and AI-assisted peptide design was performed with RFdiffusion and ProteinMPNN.

**Results:**

We identified 1,289 prostate cancer-specific G4 structures, predominantly in promoter regions. Machine learning and immune enrichment analysis pinpointed IKBKB as a key hub gene in prostate cancer progression. IKBKB was overexpressed in prostate cancer tissues, correlated with advanced stage and poor prognosis, and was regulated by promoter G4 structures via transcription factors AR and ERG. IKBKB promoted genome instability, tumor stemness, and immune microenvironment remodeling. G4 stabilization increased IKBKB expression and activated the NF-κB pathway, enhancing cancer cell viability, proliferation, and migration. Computational screening confirmed IKBKB’s druggability and identified potential inhibitors (e.g., Auranofin). AI-assisted design generated peptide inhibitors targeting IKBKB and a CRISPR-dCas9 strategy for G4 disruption.

**Conclusions:**

IKBKB is a G4-regulated, immune-related driver of prostate cancer progression. Its overexpression is linked to NF-κB activation, genomic instability, and immune microenvironment alterations. The study proposes two novel therapeutic strategies: G4 disruption at the IKBKB promoter and AI-designed peptide inhibitors. These findings provide a framework for combining epigenetic targeting with immunotherapy in prostate cancer.

## Introduction

Prostate cancer is one of the most common malignancies affecting men worldwide (1). Although localized disease can be managed effectively, advanced and metastatic prostate cancer—particularly castration-resistant prostate cancer—remains a major clinical challenge (2). Current treatments, including androgen deprivation therapy, chemotherapy, and next-generation hormonal agents, frequently lead to therapeutic resistance (3). Furthermore, prostate cancer has traditionally been regarded as an immunologically “cold” tumor with poor responses to immune checkpoint inhibitors (4, 5). The dual challenges of therapeutic resistance and immune evasion underscore the urgent need to identify novel molecular drivers and develop innovative therapeutic strategies.

G-quadruplexes (G4s) have emerged as important non-canonical nucleic acid structures with significant regulatory potential in cancer biology (6, 7). These stable four-stranded structures form in guanine-rich regions of DNA and RNA through the stacking of G-tetrads stabilized by Hoogsteen hydrogen bonding (8). G4s are not randomly distributed throughout the genome but are strategically enriched in key regulatory regions such as gene promoters, transcriptional start sites, and telomeres (9). This specific localization enables them to function as dynamic epigenetic switches that can modulate gene expression, influence chromatin architecture, and regulate DNA replication and repair (10–12). In cancer, G4s are frequently associated with the promoters of oncogenes, where their formation and stabilization can directly impact transcriptional programs that drive tumor initiation, progression, and therapeutic resistance (13). Consequently, G4s represent promising therapeutic targets, with small molecule ligands designed to stabilize or disrupt these structures showing potential for selective modulation of oncogenic pathways.

The specific role of G4 structures in prostate cancer pathogenesis and their potential as therapeutic targets remain underexplored (14). Preliminary evidence suggests that G4-mediated regulation may affect key prostate cancer pathways, including androgen receptor signaling and DNA damage response (4, 15). However, a systematic characterization of the G4 landscape in prostate cancer, particularly its relationship with the tumor immune microenvironment, has not been conducted (4). Notably, while G4-targeting approaches have been proposed in other malignancies, their application in prostate cancer—especially in combination with emerging technologies such as CRISPR-based epigenome editing and artificial intelligence (AI)—represents a largely untapped therapeutic frontier.

To address these gaps, this study employs an integrated multi-omics and computational approach. We first mapped the genome-wide G4 landscape in prostate cancer cells to identify cancer-specific G4 structures. Importantly, we prioritized the immune regulatory dimension of G4 function for three reasons: (i) prostate cancer is an immunologically “cold” tumor with limited responses to current immunotherapies, suggesting that epigenetic modifiers of immune pathways may represent critical therapeutic nodes; (ii) G4 structures are known to regulate key transcription factors and signaling molecules involved in immune modulation, yet this function remains poorly characterized in prostate cancer; and (iii) identifying G4-regulated immune-related genes could reveal druggable targets for combination epigenetic-immunotherapy strategies. By combining G4 epigenomic data with machine learning algorithms, we identified and validated key G4-regulated immune-related hub genes that drive prostate cancer progression through the NF-κB pathway. Focusing on the highest-confidence target, IKBKB, we elucidated its G4-dependent regulatory mechanism and multifaceted oncogenic functions. Finally, we leveraged this mechanistic understanding to propose two innovative therapeutic strategies: a CRISPR-dCas9-based approach for targeted G4 disruption in the IKBKB promoter, and an AI-assisted platform for the *de-novo* design of inhibitory peptides targeting the IKBKB protein. This work establishes a novel G4-regulated axis in prostate cancer progression and provides a translational framework for mechanistically informed, epigenetically targeted therapies.

## Results

### Identification of prostate cancer-specific G4 structure-associated genes

**Figure 1:**
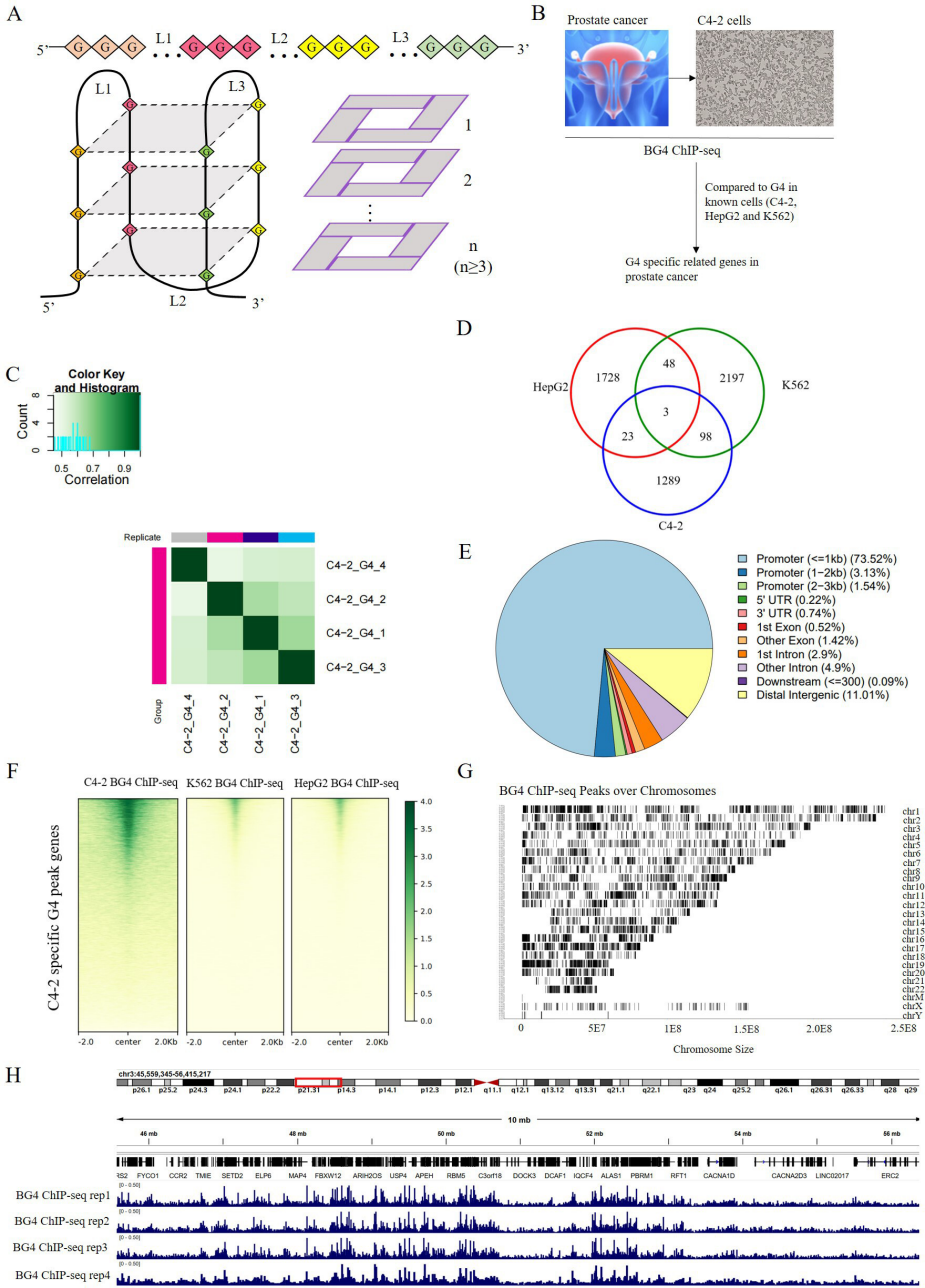
Widespread distribution of G4 structures in the PRAD cell line C4-2. **(A)** G4 structure model. **(B)** BG4 ChIP-seq sequencing. **(C)** Correlation analysis among four replicates of BG4 ChIP-seq in C4–2 cells. **(D)** Venn diagram comparing different BG4 ChIP-seq datasets. **(E)** The distribution of C4–2 G4s on different genomic regions. **(F)** BG4 ChIP-seq signals were accumulated around C4–2 G4 genes. **(G)** C4–2 BG4 ChIP-seq peaks on different chromosomes. **(H)** Representative BG4 ChIP-seq signal profiles visualized in IGV.

G-quadruplexes (G4s) are nucleic acid secondary structures that form in guanine-rich DNA or RNA sequences. Their four-stranded architecture comprises a stack of two or more planar G-tetrads, each formed by four guanines through Hoogsteen hydrogen bonding (**Figure 1A**). We identified G4 structures in the prostate cancer cell line C4–2 using BG4 ChIP-seq (**Figure 1B**). Analysis of four replicates showed correlation values ranging from 0.5 to 0.9 (**Figure 1C**), confirming high reproducibility. The recurrent G4 peaks in C4–2 cells were compared with known G4 profiles in HepG2 and K562 cell lines, identifying 1289 prostate cancer-specific G4 structures within 1303 gene regions (**Figure 1D**, **Supplementary Table 1**).

Genomic annotation revealed that C4–2 G4s are predominantly located in gene promoters and regulatory regions (**Figure 1E**). Promoter-associated peaks accounted for 78.19% of the total, with the majority (73.52%) residing within 1 kb upstream of the transcription start site (TSS), followed by 3.13% at 1–2 kb and 1.54% at 2–3 kb from the TSS. Consistent with this, BG4 signals were significantly enriched around TSSs (**Figure 1F**). G4 peaks were distributed across all chromosomes but showed notable clustering in gene-rich regions (**Figure 1G**), with the highest densities on chromosomes 1 and 2. Visualization of raw sequencing data in IGV confirmed specific and reproducible G4 signals at representative loci (**Figure 1H**). Collectively, these findings demonstrate the extensive and non-random distribution of G4 structures in C4–2 prostate adenocarcinoma cells.

### Unbiased pathway enrichment and identification of immune-related hub genes using machine learning

**Figure 2:**
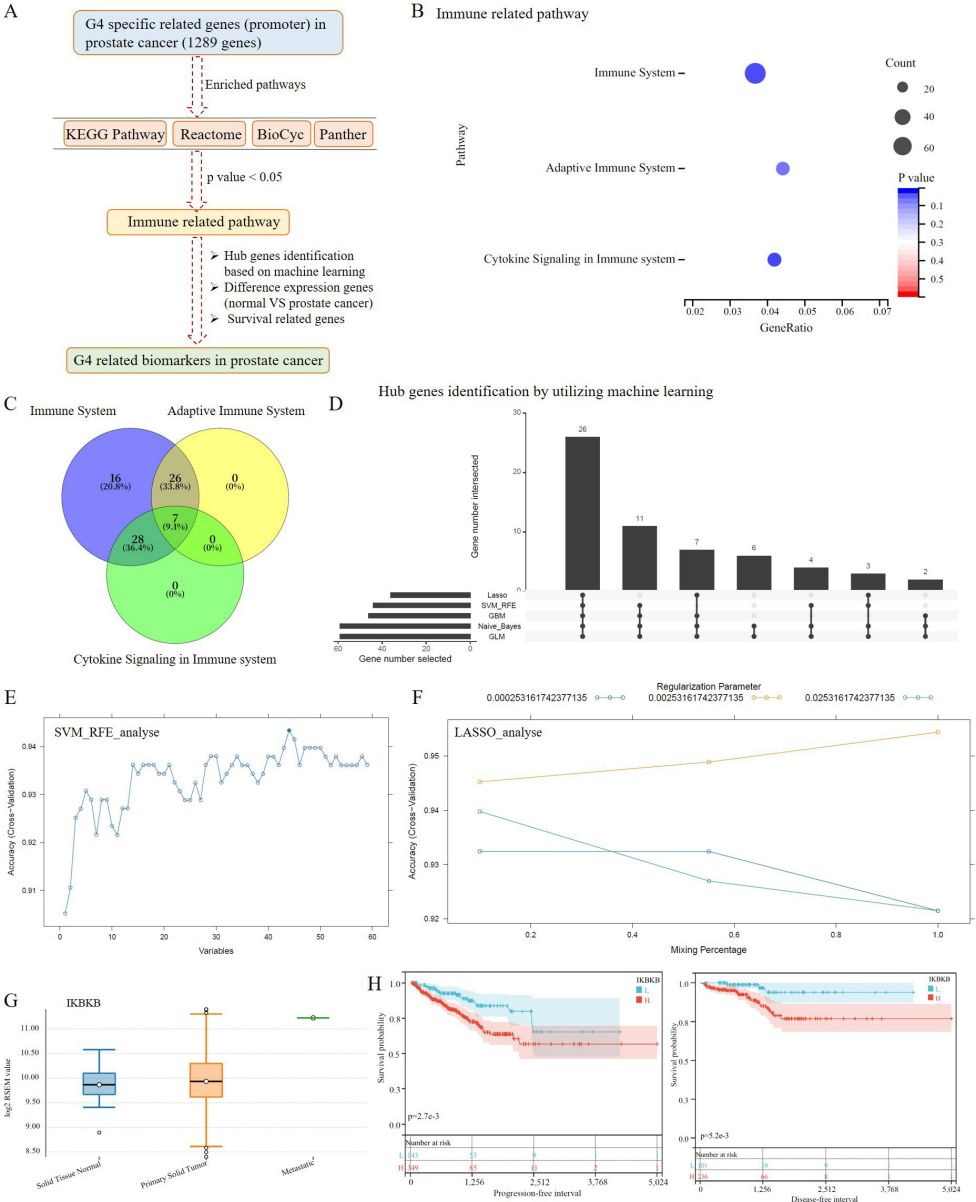
Unbiased pathway enrichment and hub immune-related gene identification using machine learning. **(A)** Workflow integrating expression matrices, machine learning, and clinical data to identify key genes. **(B)** Top enriched pathways (including immune-related and cancer hallmark pathways) for the 1,289 G4-associated genes. **(C)** Intersection genes across three immune-related pathways. **(D)** Hub genes identified by the intersection of five machine learning methods. **(E, F)** Accuracy values for SVM-RFE **(E)** and LASSO **(F)**. **(G)** IKBKB expression in para-cancerous, primary cancer, and metastatic cancer tissues. **(H)** Progression-free and disease-free survival curves for IKBKB in prostate cancer patients.

To ensure an unbiased analysis, we first performed comprehensive pathway enrichment of all 1,289 G4-associated genes using KEGG, Reactome, BioCyc, and Panther databases (**Figure 2A**). The predominant pathways enriched by these genes included not only immune-related processes (e.g., cytokine signaling, adaptive immune system, and antigen presentation) but also cancer hallmark pathways such as cell proliferation, apoptosis, DNA damage response, signal transduction, and metabolism (**Figure 2B**, **Supplementary Table 2**). This unbiased enrichment confirmed that immune-related pathways represent a major functional category among G4-regulated genes in prostate cancer, justifying our subsequent immune-focused analysis. We then set the 1,289 genes as input features for functional enrichment, machine learning, differential expression, and survival analysis (**Figure 2A**). Three immune-related pathways—the immune system, the adaptive immune system, and cytokine signaling in the immune system—showed significant enrichment (**Figure 2B**). A total of 77, 33, and 35 genes were enriched in these three pathways, respectively, with 61 genes appearing across multiple pathways (**Figure 2C**, **Supplementary Table 2**). These 61 genes were subjected to five machine learning algorithms: LASSO, SVM-RFE, GBM, Naive Bayes, and GLM. The intersection of these methods identified 26 hub genes (**Figure 2D**).

All five machine learning methods achieved accuracy values above 0.93, with SVM-RFE and LASSO exceeding 0.94 (**Figures 2E, F**). Intersection of the 26 candidate genes with differentially expressed genes in prostate cancer yielded four genes: IKBKB, HECTD2, SH3RF1, and BOLA2B (**Figure 2G**). Among these, only IKBKB was significantly associated with patient survival (**Figures 2G, H**). IKBKB expression increased with cancer progression, and high expression correlated with poorer progression-free and disease-free survival. These results identify IKBKB as a prognostic hub gene in prostate cancer.

### IKBKB is a clinically relevant immune-related gene in prostate cancer

**Figure 3:**
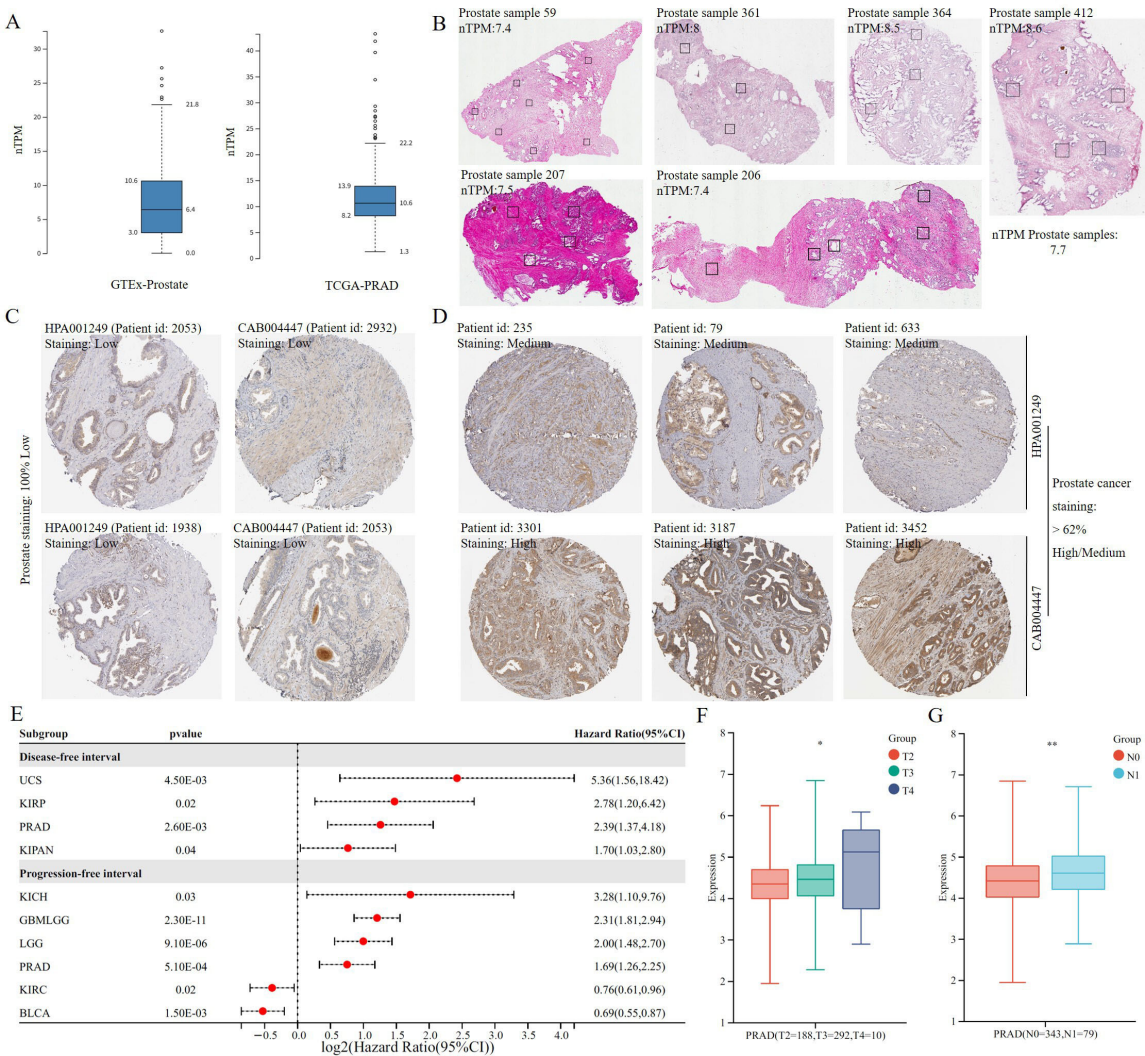
IKBKB acts as a hazard factor in prognosis of prostate cancer patients. **(A)** The IKBKB expression in normal prostate (GTEx) and PRAD (TCGA). **(B)** The IKBKB expression in normal prostate based on HPA database. **(C)** and **(D)** represent IKBKB antibody staining level in normal prostate **(C)** and cancer tissues **(D)**. **(E)** HR (hazard ratio) values for IKBKB in pan-cancer with a significance *p*-value. **(F)** and **(G)** represent IKBKB expression in different clinical stages for prostate cancer patients, that is, T clinical grades **(F)** and N grades **(G)**. * and ** represent *p*-value < 0.05 and < 0.01.

To further characterize IKBKB expression, we analyzed gene and protein levels in normal and prostate cancer tissues using GTEx, TCGA, and HPA data. The mean nTPM values for IKBKB were 6.4 in normal prostate (GTEx: 3.0–10.6) and 10.6 in prostate adenocarcinoma (TCGA-PRAD: 8.2–13.9) (**Figure 3A**). The mean nTPM value in the HPA dataset was 7.7 (5.5–10.1) (**Figure 3B**). IKBKB antibody staining (HPA001249 and CAB004447) was low in all normal prostate tissues (**Figure 3C**) but high or medium in over 62% of prostate cancer tissues (**Figure 3D**). These results demonstrate that IKBKB is overexpressed at both the transcript and protein levels in prostate cancer compared to normal tissues.

To explore the clinical significance for high IKBKB expression in prostate cancer patients, the HR (hazard ratio) values for it in pan-cancer were calculated based on Cox proportional hazards model. Then, IKBKB was found that it was a hazard factor in the prognosis of four and six types of cancer patients based on disease-free interval and progression-free interval analysis, in respectively (**Figure 3E**). It was found that only for the prognosis of prostate cancer patients, IKBKB acts as a hazard factor both in disease-free interval and progression-free interval analysis (**Figure 3E**). Furthermore, based on compared among IKBKB expression in different stages (T and N) of prostate cancer patients, it was found that as the clinical stage grade of prostate cancer increased, its expression level also rose (**Figures 3F, G**). These results indicated that IKBKB is a survival hazard factor for prostate cancer patients.

### Promoter G4 structures regulate IKBKB transcription through recruitment of AR and ERG

**Figure 4:**
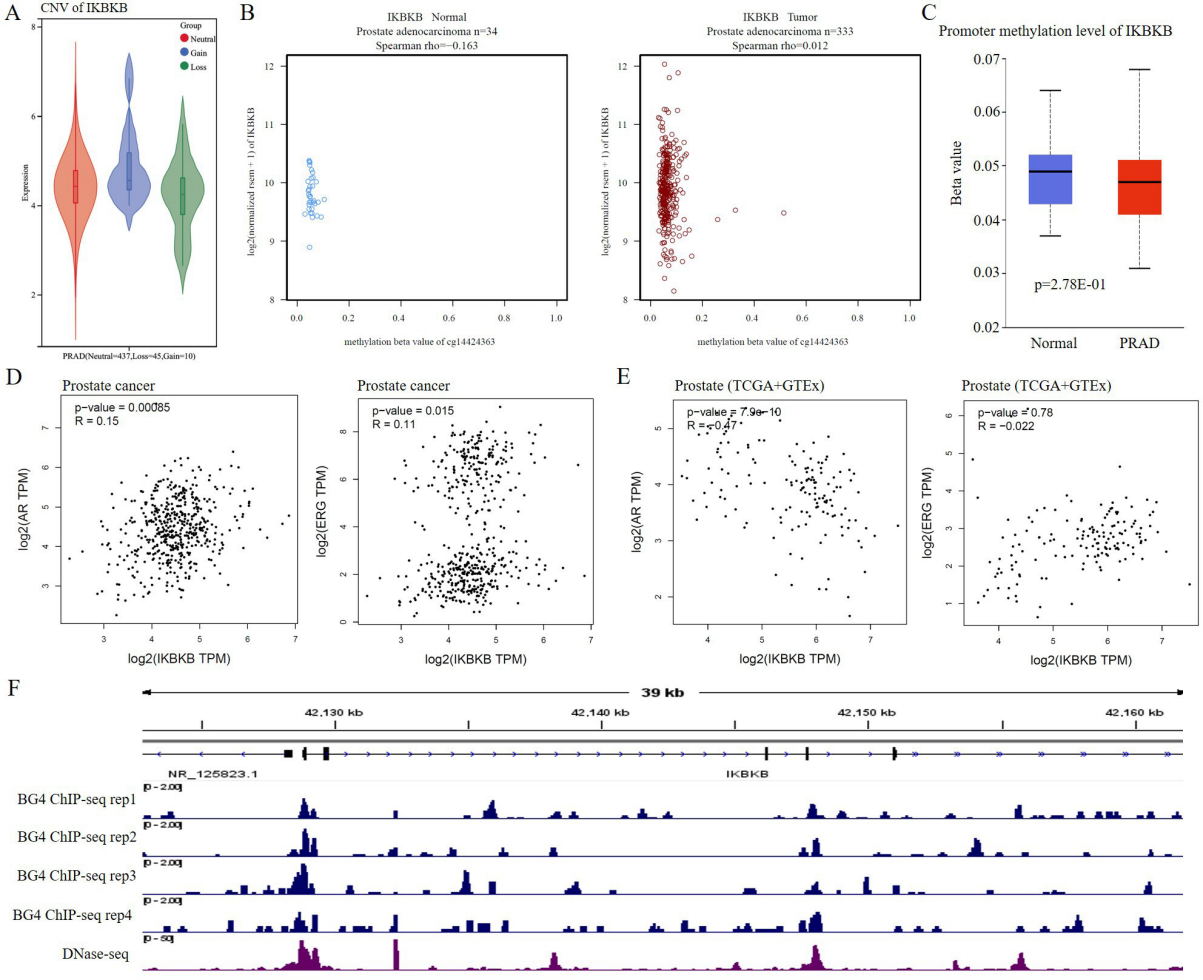
Promoter G4 affects IKBKB transcriptional activity. **(A)** CNV of IKBKB in PRAD. **(B)** Methylation beta value of probe in normal and PRAD. **(C)** Promoter methylation level of IKBKB in normal and PRAD. **(D, E)** The expression level correlation coefficient between IKBKB and transcription factors (AR and ERG) in normal **(D)** and prostate cancer tissues **(E)**. **(F)** G4 created an open region in the IKBKB promoter region, resulting in high transcription.

To elucidate the mechanisms underlying IKBKB overexpression, we examined copy number variation (CNV), promoter methylation, point mutation, histone modification, and G4 structures. CNV did not significantly affect IKBKB expression in PRAD, as no significant differences were observed among normal, gain, and loss groups (**Figure 4A**). Similarly, no significant Spearman correlation was found between IKBKB expression and methylation beta values of promoter probes in PRAD (**Figure 4B**), and no significant difference in promoter methylation levels was detected between normal and tumor tissues (**Figure 4C**). Furthermore, the point mutation frequency of IKBKB in PRAD was very low, with no evidence supporting its role in expression regulation (**Supplementary Figure 1**). Collectively, these results indicate that CNV, promoter methylation, and point mutation—the three most common mechanisms driving oncogene expression—do not account for IKBKB overexpression in prostate cancer.

To verify IKBKB transcriptional activity, we analyzed histone acetylation levels in LNCaP and PC3 prostate cancer cells. Using datasets GSE114737 and GSE137207, H3K27ac peaks were identified at the transcription start site and within the gene body of IKBKB (**Supplementary Figure 2**), confirming high transcriptional activity of IKBKB in prostate cancer.

Analysis of the GSE83653 dataset identified active transcription factors that interact with IKBKB in prostate cancer, including AR and ERG (**Supplementary Figure 2**). These transcription factors showed a positive correlation with IKBKB expression in prostate cancer but not in normal prostate tissues (**Figures 4D, E**). Furthermore, DNase-seq data demonstrated that G4 creates an open chromatin region in the IKBKB promoter, facilitating active transcription (**Figure 4F**). We precisely mapped the G4 peak (chr8:42,126,820–42,130,820) associated with IKBKB using our BG4 ChIP-seq data. This peak is located in the proximal promoter region of IKBKB under the hg38 reference genome and contains classic G4 motifs. These results suggest that the G4 structure within the IKBKB promoter enhances transcriptional activity, potentially by recruiting transcription factors such as AR and ERG, thereby contributing to IKBKB overexpression in prostate cancer.

### IKBKB is associated with core cancer hallmarks in prostate cancer

**Figure 5:**
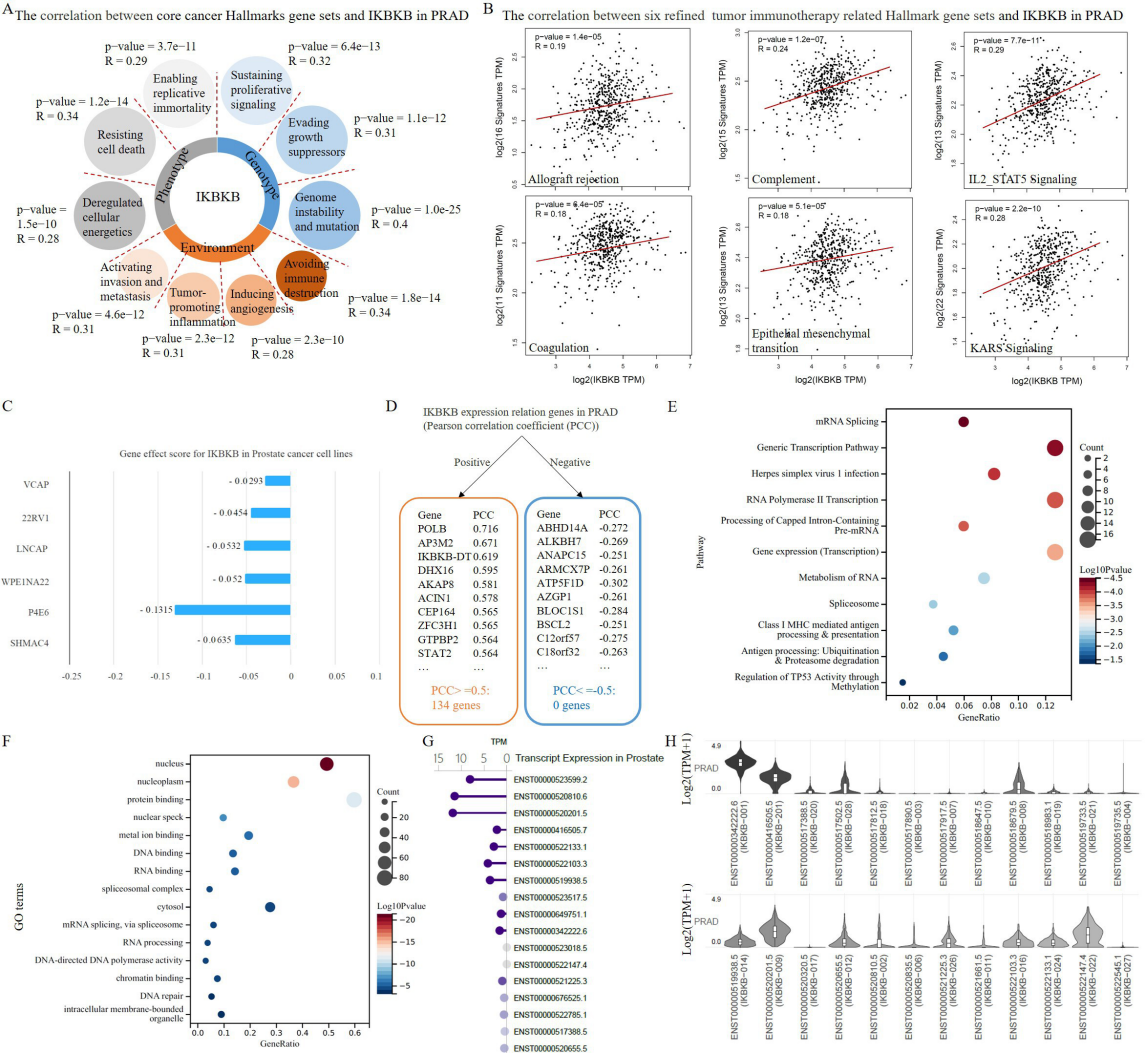
IKBKB functions in prostate cancer. **(A)** The correlation between core cancer Hallmarks gene sets and IKBKB in PRAD. **(B)** The correlation between six refined tumor immunotherapy-related Hallmark gene sets and IKBKB in PRAD. **(C)** Gene effect scores for IKBKB in prostate cancer cells. **(D)** IKBKB expression related genes in PRAD. **(E)** and **(F)** represent enriched items for KEGG pathway **(E)** and GO **(F)**. **(G)** and **(H)** represent main isoforms of IKBKB in normal and prostate cancer tissues.

To comprehensively characterize IKBKB function in prostate cancer, we calculated correlations between core cancer hallmark gene sets and IKBKB expression in PRAD. IKBKB expression showed the strongest positive correlation with genes related to immune function (R = 0.34, *p* = 1.8 × 10^-^¹^4^) and genome instability and mutation (R = 0.40, *p* = 1.0 × 10^-^²^5^) (**Figure 5A**). We also examined correlations between IKBKB and six refined tumor immunotherapy-related hallmark gene sets. IKBKB was significantly correlated with multiple immunotherapy pathways, including KRAS signaling and IL2-STAT5 signaling (**Figure 5B**). These results indicate that IKBKB represents a promising candidate drug target for PRAD and that its inhibitors may have synergistic effects when combined with immunotherapy.

To further characterize IKBKB function in PRAD, we analyzed gene effect scores and IKBKB expression-correlated genes. Gene effect scores from CRISPR knockout screens showed that IKBKB knockout inhibited prostate cancer cell growth or induced cell death (**Figure 5C**). Among IKBKB-correlated genes, 134 showed positive correlations (|PCC| > 0.5), and none showed negative correlations (**Figure 5D**, **Supplementary Table 3**). These genes were primarily enriched in transcriptional processes, including mRNA splicing (**Figures 5E, F**). Thus, the association between IKBKB and genome instability may be partially mediated through alternative mRNA splicing. Furthermore, IKBKB isoform usage differed between normal and prostate cancer tissues (**Figures 5G, H**), consistent with its potential role in regulating alternative splicing.

**Figure 6:**
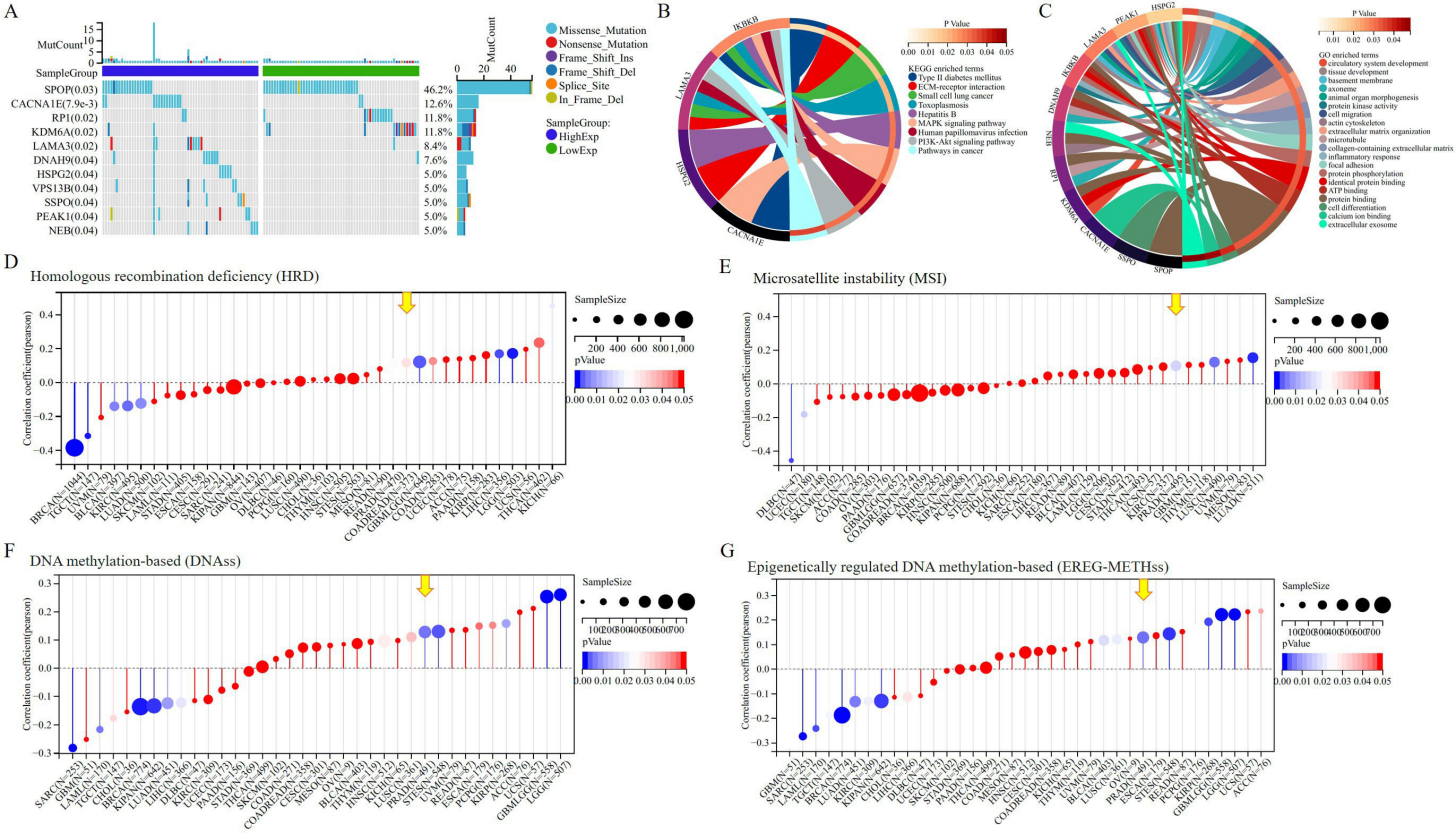
IKBKB influence genome instability and mutation in PRAD. **(A)** Differentially mutated genes in the high and low expression groups of the IKBKB in prostate cancer. **(B)** and **(C)** represent enriched items of KEGG **(B)** and GO **(C)** for differentially mutated genes. **(D)** and **(E)** indicate correlation coefficient analysis between IKBKB expression and HRD **(D)**/MSI **(E)** in PRAD. **(F)** and **(G)** represent correlation coefficient analysis between IKBKB expression and DNAss **(F)**/EREG-METHss **(G)** in PRAD.

Given IKBKB’s strong correlation with genome instability and mutation (**Figure 5A**), we further investigated its association with gene mutations, genomic heterogeneity, and tumor stemness in PRAD. Eleven genes showed significantly different mutation frequencies between IKBKB high- and low-expression groups (**Figure 6A**). These genes were enriched in MAPK signaling, PI3K-AKT signaling, and other cancer-related pathways (**Figures 6B, C**), suggesting that IKBKB may influence cancer progression by modulating mutation patterns in oncogenic genes. IKBKB expression also correlated positively with genomic heterogeneity markers, including homologous recombination deficiency (HRD, R = 0.11, *p* = 0.02, **Figure 6D**) and microsatellite instability (MSI, R = 0.11, *p* = 0.01, **Figure 6E**). Furthermore, IKBKB expression positively correlated with tumor stemness indices: DNAss (R = 0.13, *p* = 0.004, **Figure 6F**), EREG-METHss (R = 0.13, *p* = 0.004, **Figure 6G**), EREG-EXPss (R = 0.13, *p* = 0.003, **Supplementary Figure 3**), and ENHss (R = 0.11, *p* = 0.007, **Supplementary Figure 4**). These results demonstrate that IKBKB functions as a multifaceted driver of prostate cancer progression, extending beyond immune regulation to genome instability and tumor stemness.

### IKBKB promotes the alteration of the immune microenvironment of prostate cancer

**Figure 7:**
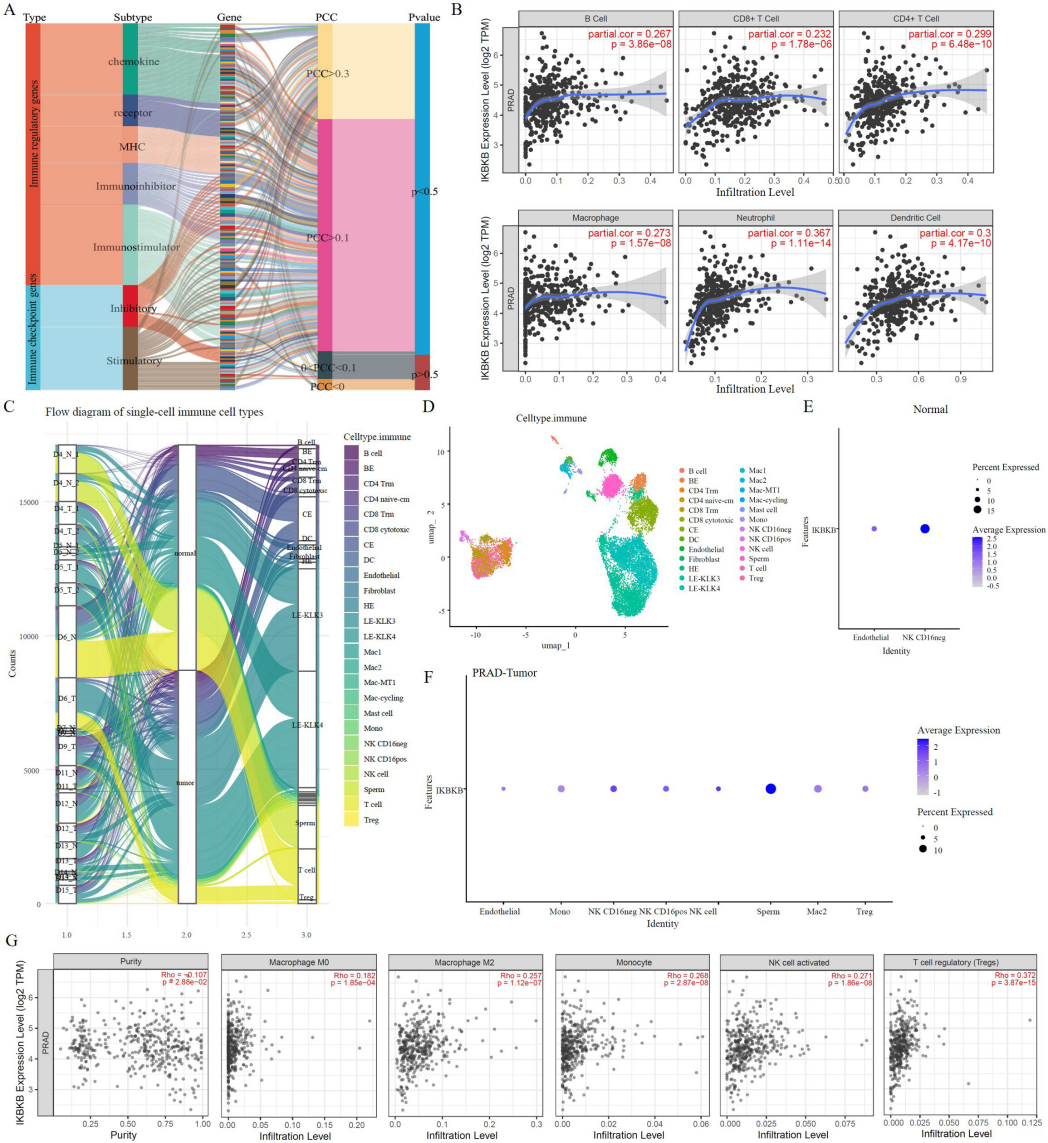
IKBKB functions in immune microenvironment for prostate cancer. **(A)** The mRNA expression correlation between IKBKB and immune regulatory/checkpoint genes in PRAD. **(B)** The correlation between IKBKB expression and immune infiltration levels of main immune cell types. **(C)** The composition of immune cells in normal and tumor for prostate. **(D)** Then umap for cell types in scRNA. **(E)** and **(F)** represent IKBKB high expression cell types. **(G)** IKBKB promotes the infiltration level of immune cells that are highly expressed in prostate cancer.

To explore IKBKB’s immune functions and potential synergy between its inhibition and immunotherapy, we calculated correlations between IKBKB and immune regulatory/checkpoint genes in PRAD. Immune regulatory genes were classified into five subtypes (chemokine, receptor, MHC, immunoinhibitor, and immunostimulator), and immune checkpoint genes into two subtypes (inhibitory and stimulatory) (**Figure 7A**, **Supplementary Table 4**). IKBKB showed positive correlations with multiple immune regulatory and checkpoint genes, with some correlation coefficients exceeding 0.3 (**Figure 7A**). Using the TIMER method, we found that IKBKB expression positively correlated with infiltration levels of multiple immune cell types in PRAD, including B cells (R = 0.27), CD8+/CD4+ T cells (R = 0.23/0.30), macrophages (R = 0.27), neutrophils (R = 0.37), and dendritic cells (R = 0.30) (**Figure 7B**). These results suggest that IKBKB influences the immune microenvironment of prostate cancer.

To characterize IKBKB’s role in the immune microenvironment, we downloaded and analyzed single-cell RNA-seq data from prostate cancer (EGAS00001005787). Based on previous classification, the composition of immune cells in normal and tumor prostate tissues is shown in **Figure 7C**. Immune cell counts were substantially lower than non-immune cells in prostate cancer (**Figures 7C, D**). Compared to normal tissues, IKBKB was expressed in a broader range of immune cell types in prostate cancer (**Figures 7E, F**, **Supplementary Figures 5**, **6**). In normal tissues, IKBKB was highly expressed in endothelial and NK CD16-negative cells. In prostate cancer, IKBKB was highly expressed in monocytes, NK cells (CD16-negative and CD16-positive), regulatory T cells, and macrophages, as well as in endothelial cells (**Figures 7E, F**). Furthermore, IKBKB expression promoted infiltration of these immune cell types in prostate cancer (**Figures 7F, G**), including potential involvement in M2 macrophage polarization (**Figure 7G**). These results indicate that IKBKB plays an important role in remodeling the tumor immune microenvironment in PRAD.

### G4-mediated IKBKB upregulation activates NF-κB signaling and promotes malignant phenotypes

**Figure 8:**
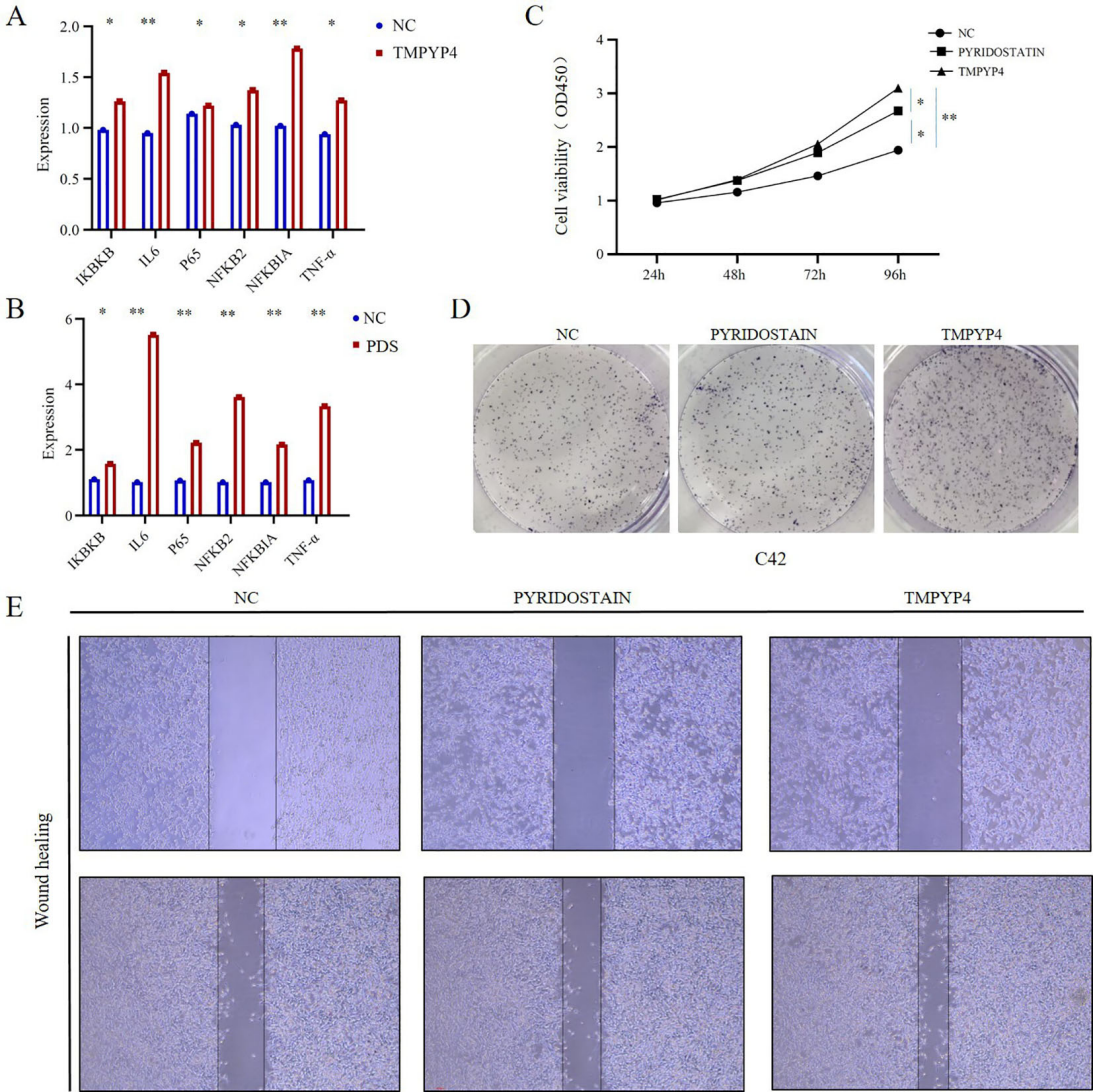
IKBKB mediates the effects of G4 stabilization on prostate cancer cell malignancy. **(A, B)** Relative mRNA levels of IKBKB and key NF-κB signaling genes in C4–2 cells treated with G4 stabilizers TMPY4 **(A)** and PYRIDOSTATIN (PDS) **(B)** for 24h, as determined by qRT-PCR. NC represents the negative control. **(C)** Activate IKBKB could promotes prostate cancer cell viability. Cell viability of C4–2 cells was assessed by CCK-8 assay at the indicated time points (24, 48, 72, 96h). Absorbance was measured at OD450. **(D)** G4 activates IKBKB could enhances prostate cancer cell proliferation. Representative images of colony formation assays in C4–2 cells after treatments. Colonies were stained and counted after 10–14 days. **(E)** IKBKB could accelerates prostate cancer cell migration. Representative images of wound healing assays in C4–2 cells after treated with G4 stabilizers. Wound closure was monitored and measured at 0 and 24h.* and ** represent *p* < 0.05 and 0.01, respectively.

To clarify the functional impact of G4 stabilization on IKBKB, we treated C4–2 cells with the G4 stabilizers TMPY4 and PYRIDOSTATIN (PDS). In preliminary experiments, lower concentrations (5–20 nM) of G4 stabilizers failed to induce significant changes in IKBKB expression, consistent with reports that low concentrations of G4 ligands are insufficient to stabilize G4 structures and effectively regulate target gene expression. qPCR analysis revealed that treatment with G4 stabilizers elevated mRNA levels of IKBKB, IL6, P65 (RELA), NFKB2, NFKBIA, and TNF-α compared to pre-treatment levels (**Figures 8A, B**). The magnitude of IKBKB upregulation induced by G4 stabilization was comparable to the differential observed between normal and disease states. Using 50 nM TMPY4 and PDS, statistical analysis of OD450 values at 24, 48, 72, and 96h demonstrated consistent effects on prostate cancer cell viability (**Figure 8C**). CCK−8 assays confirmed that the IC50 of PDS in C4−2 cells exceeds 20 µM (**Supplementary Figure 7**), and cell viability at 50 nM remained above 100% relative to control, with no significant cytotoxicity. These results indicate that IKBKB upregulation upon 50 nM PDS treatment reflects a specific effect of G4 stabilization rather than non-specific cellular stress.

Colony formation assays showed that G4-stabilization-induced IKBKB upregulation promotes prostate cancer cell proliferation (**Figure 8D**). Wound healing assays further demonstrated that IKBKB upregulation enhances cell migration (**Figure 8E**). Conversely, IKBKB knockdown significantly inhibited both proliferation and migration (**Supplementary Figure 8**). Collectively, these results suggest that IKBKB promotes viability, proliferation, and migration of prostate cancer cells via the NF-κB signaling pathway.

The IKBKB promoter harbors a clear G4 peak in C4−2 (prostate cancer) cells based on our own BG4 ChIP−seq data, whereas in HepG2 (hepatocellular carcinoma) cells, publicly available BG4 ChIP−seq data showed no significant G4 signal in the IKBKB promoter. We then compared the basal expression levels of IKBKB in these two cell lines using HPA RNA−seq data. The average expression of IKBKB in C4−2 cells was 7.5 nTPM, significantly higher than that in HepG2 cells (5.3 nTPM). This observation suggests that the presence of a promoter G4 structure is associated with higher basal transcriptional activity of IKBKB. Meanwhile, **Figures 8A, B** shown that pharmacological stabilization of G4 enhances IKBKB expression. This functional intervention directly demonstrates that stabilizing the G4 structure enhances IKBKB transcription, further supporting a positive regulatory role of G4 on IKBKB. Taken together: (a) under native conditions, the cell line with a promoter G4 (C4−2) shows higher IKBKB expression than the cell line without it (HepG2); (b) artificial stabilization of G4 further increases IKBKB expression. Although these lines of evidence cannot fully replace classical G4−mutant reporter assays, they provide strong supportive evidence that the promoter G4 structure promotes IKBKB transcription to affect its functions promoting cancer progression in PRAD.

### Computer-assisted small molecule drug screening for IKBKB

**Figure 9:**
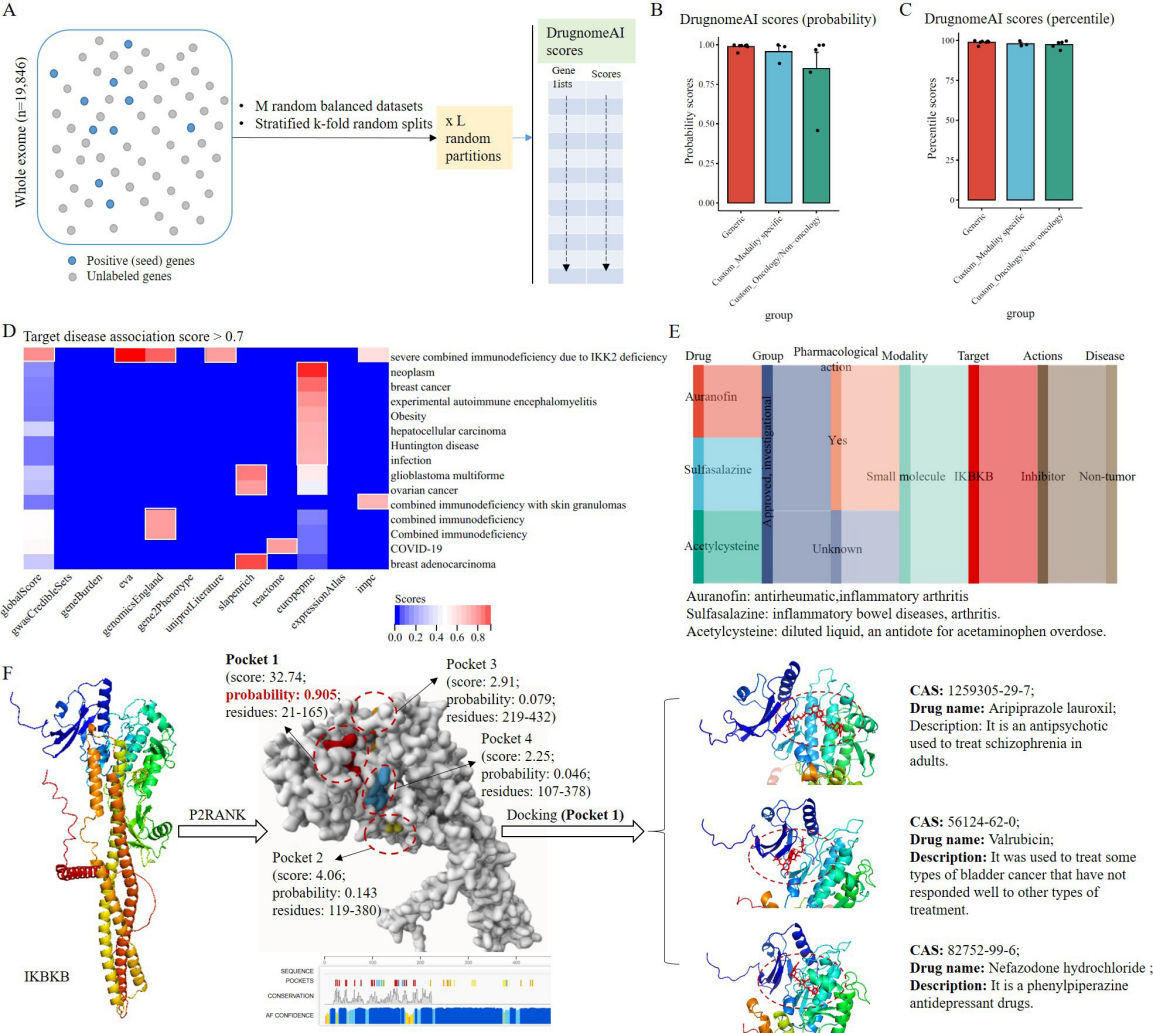
IKBKB druggability scores and it potential small molecule inhibitors. **(A)** The workflow of DrugnomeAI. The whole exome is split into random balanced subsets of positive (i.e., druggable) and unlabelled (i.e., druggability is unknown) genes. An ensemble of classifiers is generated such that multiple models are trained on each subset with stratified tenfold cross-validation. The process is repeated for L stochastic iterations. The final druggability scores are obtained by averaging the prediction scores from out-of-bag sets across all stochastic iterations from the ensemble models. The DrugnomeAI scores for probability **(B)** and percentile **(C)** for IKBKB. **(D)** Disease association for IKBKB. **(E)** Drugs targeting IKBKB in DrugBank. **(F)** Computer-assisted small molecule drugs screening based on IKBKB structure and FDA approved drugs.

To assess IKBKB as a clinical drug target, we utilized DrugnomeAI, a stochastic semi-supervised machine learning framework that infers gene druggability across the human exome (19,846 genes) by integrating historical data from known drug targets with evidence on gene tractability (**Figure 9A**). The mean DrugnomeAI probability and percentile scores for IKBKB were 0.85 and 97.46, respectively (**Figures 9B, C**). Disease association analysis using the Open Targets platform revealed that IKBKB is strongly associated with immunodeficiency-related diseases and multiple cancer types (association score > 0.7) (**Figure 9D**). These results confirm IKBKB as a druggable target for prostate cancer therapy, with potential for synergistic efficacy when combined with immunotherapy.

To identify potential drugs targeting IKBKB, we queried the DrugBank database and found three approved drugs—Auranofin, Sulfasalazine, and Acetylcysteine—that target IKBKB (**Figure 9E**). All three are non-oncology drugs with established safety profiles, suggesting potential for repurposing in prostate cancer. To expand the search, we performed structure-based virtual screening of 7,623 FDA-approved small molecules against the IKBKB structure and binding pockets predicted by AlphaFold and P2Rank (**Figure 9F**). The most probable drug-binding pocket was pocket 1 (residues 21–165, probability = 0.905). Docking analysis using Discovery Studio 3.0 identified three additional candidates: Aripiprazole lauroxil, Valrubicin (an oncology drug), and Nefazodone hydrochloride. These results indicate that multiple approved non-oncology drugs may inhibit IKBKB, supporting drug repurposing strategies for prostate cancer therapy.

### AI-assisted design of G4 disruptors and peptide inhibitors targeting IKBKB

**Figure 10:**
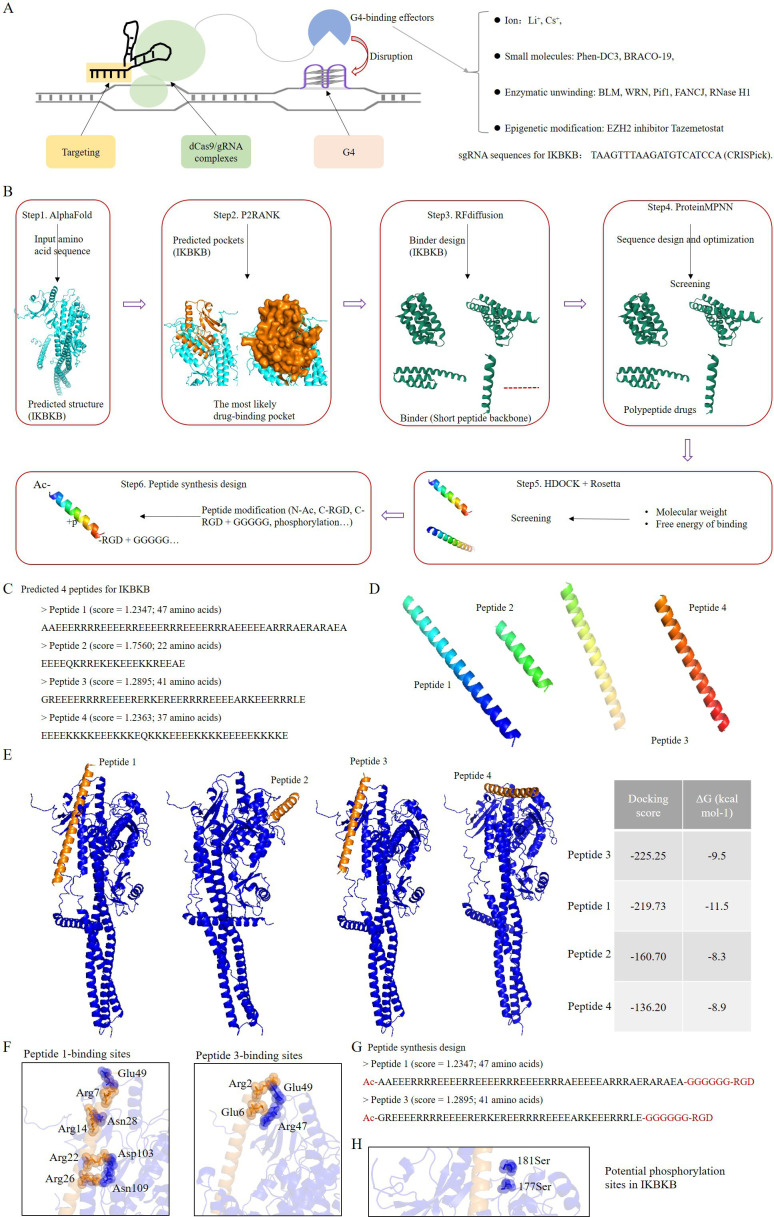
IKBKB promoter G4 disrupters and peptide drugs design and refinement. **(A)** The G4-binding effectors fused to dCas9 are used to target G4 in the specific genome loci (IKBKB promoter G4). **(B)** The workflow of peptide design and refine based on AI tools. It is consist of six steps: (i) 3d structure construction; (ii) binding pockets prediction; (iii) binder design; (iv) sequence design and optimization; (v) peptide segment screening; (vi) peptide synthesis design. **(C)** Four predicted peptide sequences for IKBKB. The small score represents the better peptide for drug design. **(D)** 3D structure for four predicted peptides. **(E)** The complex structure for each peptide and IKBKB and their corresponding docking assessment scores. **(F)** The binding sites for peptides (peptides 1 and 3) and IKBKB. **(G)** Peptide synthesis design for peptide 1/3 of IKBKB. Ac- represent N terminal acetylation of a peptide. –RGD represent Arg-Gly-Asp sequence is added in the C terminal of the peptide. Score represent the RFdiffusion score. **(H)** Potential phosphorylation sites in IKBKB.

Since promoter G4 is a key regulator of IKBKB expression in PRAD, disrupting this G4 represents a novel therapeutic strategy. Previous studies have used G4-stabilizing proteins or ligands with CRISPR to facilitate targeted G4 folding at specific genomic loci. Here, we developed the converse approach: employing G4-binding effectors with CRISPR-dCas9 to disrupt G4 folding at the IKBKB promoter (**Figure 10A**). The G4-binding effectors include four types: ions, small molecules, enzymatic unwinding factors, and epigenetic modifiers. The predicted sgRNA sequence is: TAAGTTAAGATGTCATCCA. This refined approach enables targeted inhibition of IKBKB transcription by disrupting promoter G4 structures.

Since potential side effects of small molecule drugs and G4-binding effectors, peptide-based therapies have attracted considerable interest in the treatment of cancer due to their promising therapeutic properties and enhanced safety profiles. In this study, we developed a method for design and refine peptide drugs for cancer potential based on druggability targets (**Figure 10B**). Firstly, the 3d structure of IKBKB was predicted based on AlphaFold (**Figures 9F**, **10B**). Secondly, binding pockets for IKBKB were predicted based on P2Rank which is formed by machine learning method (**Figures 9F**, **10B**). Thirdly, binder design model in RFdiffusion, which utilize *de-novo* protein design seeks to generate proteins with specified structural, was utilized to construct peptide backbone (**Figure 10B**). Fourth, peptide sequences predicted by utilizing ProteinMPNN, which is a method that uses a message passing neural network (MPNN) to design proteins with desired properties (**Figures 10B–D**). Fifth, the complex of peptide construction and assessment based on HDOCK and Rosetta (**Figures 10B, E**). Finally, peptide synthesis design, such as N-AC, C-GGGGGG-RGD for increasing the flexibility of the polypeptide in reaching the cell surface and enhance the possibility of binding with integrin (**Figures 10E–G**). Based on these six steps, we obtain the two optimized peptides, that is, peptide 1 (Ac-AAEEERRRREEEERREEEERRREEEERRRA EEEEEARRRAERARAEA-GGGGGG-RGD) and peptide 3 (Ac-GREEEERRRRE EEERERKEREERRRREEEEARKEEERRRLE-GGGGGG-RGD). Then, we found they interaction sites in pocket 2 domain (**Figures 9F**, **10F**) which is around the potential phosphorylation sites in IKBKB (**Figure 10H**). Therefore, these potential polypeptide drugs probably by blocking the phosphorylation of the IKBKB site through spatial steric hindrance, the activity expression of IKBKB is inhibited, thereby preventing its functional activity in prostate cancer.

## Discussions

This study elucidates the role of G-quadruplex (G4) structures in gene regulation in prostate cancer. By integrating multi-omics analysis with machine learning, we identified IKBKB as a novel G4-regulated, immune-related hub gene. Our findings expand the understanding of G4 biological functions and provide a theoretical foundation for combined targeted and immunotherapy strategies in prostate cancer.

A genome-wide map of G4 structures in the prostate cancer C4–2 cell line was constructed. The results show that G4 structures are widely and non-randomly distributed in prostate cancer cells and are significantly enriched in gene promoter regions. This aligns with the prevailing understanding that G4 structures play a key role in transcriptional regulation (11, 16–18). By comparing G4 profiles across different cell lines, we screened for prostate cancer-specific G4 structures and their associated genes, laying the groundwork for subsequent research focusing on disease-specific mechanisms.

Based on the G4-related gene set, we integrated immune pathway enrichment analysis with various machine learning algorithms (LASSO, SVM_RFE, GBM, etc.) to narrow down the candidate genes from an initial 1289 to 26 and finally pinpointed IKBKB as the hub gene most strongly associated with prostate cancer progression and prognosis. IKBKB is a key catalytic subunit of the IKK complex, central to the NF-κB signaling pathway, which is crucial in inflammation, immune response, and tumorigenesis (19, 20). Our analysis of clinical data shows that IKBKB expression is significantly higher in cancerous tissues compared to normal tissues. Furthermore, its high expression correlates with more advanced clinical stages (T/N stage) and worse prognosis (disease-free and progression-free intervals), strongly supporting IKBKB’s role as a driver of prostate cancer progression and a potential prognostic marker.

At the mechanistic level, we investigated the causes of IKBKB overexpression. Unlike many oncogenes, IKBKB upregulation is not driven by gene copy number variations, promoter hypermethylation, or frequent point mutations. Epigenetic data indicate that the IKBKB promoter region exhibits an open chromatin state in cancer cells, marked by active histone modifications such as H3K27ac. More importantly, we identified specific G4 structures within its promoter region. Building on existing research, we propose a working model: this G4 structure may alter local DNA conformation to form an open chromatin region, thereby more efficiently recruiting key prostate cancer transcription factors (such as AR and ERG) to cooperatively enhance IKBKB transcription.

Meanwhile, cross−cell line comparison shows that the presence of a promoter G4 structure correlates with higher basal IKBKB expression, and pharmacological stabilization of G4 further upregulates IKBKB transcription, collectively supporting a positive regulatory role of the promoter G4 in IKBKB expression. The current evidence is correlative and pharmacological; it does not fully substitute for classical reporter assays using G4−disrupting mutations. Despite these limitations, this provides a novel epigenetic perspective on the aberrant activation of the NF-κB pathway in prostate cancer. While G4 ligands such as PDS may have off−target effects, their consistent ability to upregulate IKBKB and activate the NF−κB pathway across independent experiments supports a functional link between promoter G4 stabilization and enhanced IKBKB transcription. Moreover, our cross−cell line comparison (C4−2 with a promoter G4 vs. HepG2 without a G4) showed higher basal IKBKB expression in the G4−positive line without any ligand treatment, partially mitigating concerns over ligand specificity and suggesting that endogenous G4 structures positively influence IKBKB expression.

Functionally, IKBKB exhibits multifaceted pro-tumorigenic properties. It shows the strongest positive correlation with core cancer hallmarks, particularly genome instability and mutation. Further analysis reveals that high IKBKB expression positively correlates with homologous recombination deficiency, microsatellite instability, and several tumor stemness indices. Moreover, IKBKB expression is significantly positively correlated with the expression of various immune checkpoint molecules and immunomodulators and the infiltration levels of immune cells (e.g., CD8+ T cells, macrophages, dendritic cells). Single-cell transcriptome analysis further reveals that within the tumor microenvironment, IKBKB is highly expressed in a broader range of immune cell types (e.g., monocytes, NK cells, and regulatory T cells). These results collectively depict a dual role for IKBKB: driving tumor evolution by exacerbating genome instability on one hand and potentially influencing immunotherapy response by remodeling the immune microenvironment on the other.

Given the pivotal role of IKBKB, we evaluated its druggability. Computational models predict IKBKB has high drug-target potential. Through virtual screening, we identified several potential IKBKB inhibitors among approved drugs (e.g., Auranofin, Sulfasalazine). More importantly, we proposed two innovative therapeutic strategies: first, using a CRISPR-dCas9 system to deliver G4-binding effector molecules specifically to disrupt the G4 structure in the IKBKB promoter, thereby inhibiting its transcription at the epigenetic level. Second, employing AI-assisted protein design platforms (such as RFdiffusion and ProteinMPNN) to design *de-novo* peptide inhibitors capable of specifically binding IKBKB and potentially blocking its phosphorylation activity. These strategies aim to achieve targeted therapy with higher specificity and lower toxicity.

In summary, this study integrates the G4 epigenetic landscape in prostate cancer with machine learning and AI-driven drug discovery to identify and validate IKBKB as a novel therapeutic target. Our work reveals the importance of the G4-IKBKB-NF-κB axis in prostate cancer progression and provides a foundation for developing combination therapeutic strategies based on G4 modulation and protein targeting, with potential applications in refractory forms such as castration-resistant prostate cancer.

## Limitations and future directions

Several limitations of this study should be acknowledged. First, while we have leveraged publicly available datasets (GTEx, TCGA, HPA, and DrugBank) for validation, these data were not generated in-house. To strengthen our findings, future studies should incorporate proprietary experimental data, including additional prostate cancer cell lines and patient-derived samples, to confirm the G4-IKBKB regulatory axis. Second, the immune-related functions of IKBKB were primarily inferred from correlation analyses of publicly available transcriptomic data. Establishing *in vivo* models—such as G4-binding effector-dCas9 knock-in mice or patient-derived xenograft (PDX) models with IKBKB knockdown—will be essential to validate the immune regulatory roles of G4-mediated IKBKB expression and to assess the efficacy of the proposed therapeutic strategies in a physiological context. Third, while our AI-assisted peptide design provides promising candidates, experimental validation of peptide binding affinity, specificity, and anti-tumor activity *in vivo* remains to be performed. Addressing these limitations in future work will be critical to translating our findings into clinically actionable therapeutic strategies.

## Conclusion

This study integrates G4 epigenomics, bioinformatics, and machine learning to systematically investigate prostate cancer. Our key findings are as follows. First, we mapped the genome-wide G4 landscape in the C4–2 prostate cancer cell line, demonstrating enrichment in promoter regions. Second, through unbiased pathway enrichment, we confirmed that immune-related pathways represent a major functional category among G4-associated genes, supporting our subsequent immune-focused analysis. Third, we identified IKBKB as a prognostically significant, immune-related hub gene with elevated expression in tumor tissues and association with poor clinical outcomes. Fourth, we elucidated a mechanism whereby the G4 structure in the IKBKB promoter establishes an open chromatin state that facilitates recruitment of transcription factors AR and ERG, driving transcriptional activation. Fifth, we demonstrated that G4 stabilization upregulates IKBKB and activates the NF-κB pathway, thereby enhancing malignant phenotypes. Finally, we confirmed the druggability of IKBKB and proposed two innovative therapeutic strategies: CRISPR-dCas9-mediated disruption of the IKBKB promoter G4 and AI-assisted design of inhibitory peptides targeting IKBKB.

This work establishes the pathogenic role of IKBKB in prostate cancer and provides a research framework spanning epigenetic feature discovery, target identification, and AI-assisted therapeutic design. These findings advance understanding of prostate cancer pathogenesis and provide a foundation for developing precision combination therapies, particularly for refractory disease. Future research should validate the proposed therapeutic strategies in *in vivo* experimental models and explore the clinical potential of combining IKBKB-targeted interventions with existing immunotherapies.

## Methods

### Cell culture and treatments

The human prostate adenocarcinoma cell line C4–2 was cultured in RPMI-1640 medium supplemented with 10% fetal bovine serum and 1% penicillin-streptomycin at 37°C in a 5% CO_2_ humidified incubator. For G4 stabilization experiments, cells were treated with 5 μM TMPY4 or 10 μM PYRIDOSTATIN for 24h. An equivalent volume of DMSO was used as the vehicle control.

### G4 structure detection by BG4 ChIP-seq

G4 structures were mapped using the BG4 antibody-based chromatin immunoprecipitation followed by sequencing (ChIP-seq). Briefly, chromatin from C4–2 cells was cross-linked, sonicated, and immunoprecipitated with the BG4 antibody. Libraries were prepared using the NEBNext Ultra II DNA Library Prep Kit and sequenced on an Illumina NovaSeq 6000 platform. Four biological replicates were performed to ensure reproducibility.

### Bioinformatic analysis of G4 data

Raw sequencing reads were aligned to the human reference genome (hg38) using Bowtie2. G4 peaks were called with MACS2 and annotated using ChIPseeker (21). Correlation between replicates was assessed using Pearson’s coefficient. G4 distribution across genomic regions and chromosomes was visualized with custom R scripts.

### Immune pathway enrichment and machine learning

Genes associated with G4 peaks were subjected to functional enrichment analysis using KEGG, Reactome, BioCyc, and Panther databases. Immune-related genes were further analyzed using five machine learning algorithms: LASSO, SVM-RFE, Gradient Boosting Machine (GBM), Naïve Bayes, and Generalized Linear Model (GLM) (22). Genes consistently selected across methods were considered hub genes.

### Clinical and expression data analysis

Gene expression data from normal prostate (GTEx) and prostate adenocarcinoma (TCGA-PRAD) were downloaded. Protein expression data were obtained from the Human Protein Atlas (HPA) (23). Survival analysis was performed using Kaplan–Meier curves and Cox proportional hazards models. Differential expression across clinical stages (T and N) was assessed using the Mann Whitney U test.

### Mechanism exploration

Copy number variation (CNV), promoter methylation (450K array), and mutation data for IKBKB were retrieved from TCGA. Correlation between IKBKB expression and transcription factor activity (AR, ERG) was analyzed using data from GSE83653. Histone modification (H3K27ac) ChIP-seq data from GSE114737 and GSE137207 were visualized in IGV.

### Functional and microenvironment analysis

Correlations between IKBKB expression and cancer hallmarks, immune checkpoint genes, and immune cell infiltration (estimated via TIMER) were computed. Single-cell RNA-seq data (EGAS00001005787) were re-analyzed to assess IKBKB expression across cell types in prostate tumor microenvironments.

### Gene expression analysis by qRT-PCR

Total RNA was extracted using TRIzol reagent. cDNA was synthesized with a reverse transcription kit. Quantitative real-time PCR was performed using SYBR Green Master Mix on a QuantStudio system. Primer sequences are listed in as following: IKBKB: CGCGTTAAGATTCCCGCATTT, ATGGCAATCTGCTCACCTGT; IL6: CCA CCGGGAACGAAAGAGAA, GAGAAGGCAACTGGACCGAA; TNF-α: TCCTTT CCAGGGGAGAGAGG, AAAACAACCCTCAGACGCCA; P65: CTTCCAAG AAGAGCAGCGTG, TTGGGGGCACGATTGTCAAA; NFKB2: AGAGGG AGGAGGGCCTTTAG, CGGGTCCGCGTATCTTTGTA; NFKBIA: TGTGCTT CGAGTGACTGACC; TCACCCCACATCACTGAACG.

### Cell viability assay

C4–2 cells were treated with 50 nM of either G4 stabilizer (TMPY4 or PDS) for 24h prior to seeding in 96-well plates. Cell viability was assessed at 24, 48, 72, and 96h by adding 10 μl of CCK-8 solution per well, incubating for 2h, and measuring absorbance at 450 nm.

### Colony formation assay

C4–2 cells were seeded at low density (500 cells/well) in six-well plates and cultured for 10–14 days. Colonies were fixed with methanol, stained with 0.1% crystal violet, and manually counted. Only colonies containing >50 cells were included in the quantification.

### Wound healing assay

C4–2 cells were seeded in 12-well plates to form a confluent monolayer. A sterile 200 μl pipette tip was used to create a linear scratch. After washing to remove debris, cells were cultured in serum-free medium. Wound closure was photographed at 0 and 24hunder a phase-contrast microscope. The relative migration distance was quantified using ImageJ software (2, 24).

### Druggability assessment and drug screening

Druggability of IKBKB was predicted using DrugnomeAI. Existing IKBKB-targeting drugs were queried from DrugBank. Structure-based virtual screening was performed using the predicted IKBKB 3D structure from AlphaFold and binding pockets from P2Rank (25–28). Molecular docking was carried out with Discovery Studio 3.0 against an FDA-approved drug library.

### AI-assisted peptide design

Peptide binders targeting IKBKB were designed using RFdiffusion for backbone generation and ProteinMPNN for sequence optimization (29). Designed peptides were docked to IKBKB using HDOCK and Rosetta (30). Final peptides were modified with N-terminal acetylation and C-terminal RGD motifs for stability and targeting.

### Statistical analysis

All statistical analyses were performed in R (v4.2.0). P-values < 0.05 were considered significant. Correlation coefficients are reported as Spearman’s rho unless stated otherwise.

## Data Availability

The original contributions presented in the study are publicly available. This data can be found here: GEO database, and the corresponding GEO accession number is GSE334282.
