# Supplementary material for: Identification of G4-regulated immune-related drug targets for prostate cancer based on G4 screen and machine learning
Source: Front Immunol. 2026 Jun 10;17:1806289. doi: 10.3389/fimmu.2026.1806289 (PMC13291070; doi:10.3389/fimmu.2026.1806289)
Supplement: Supplementary file 1 [file DataSheet1.docx]

Supplementary materials


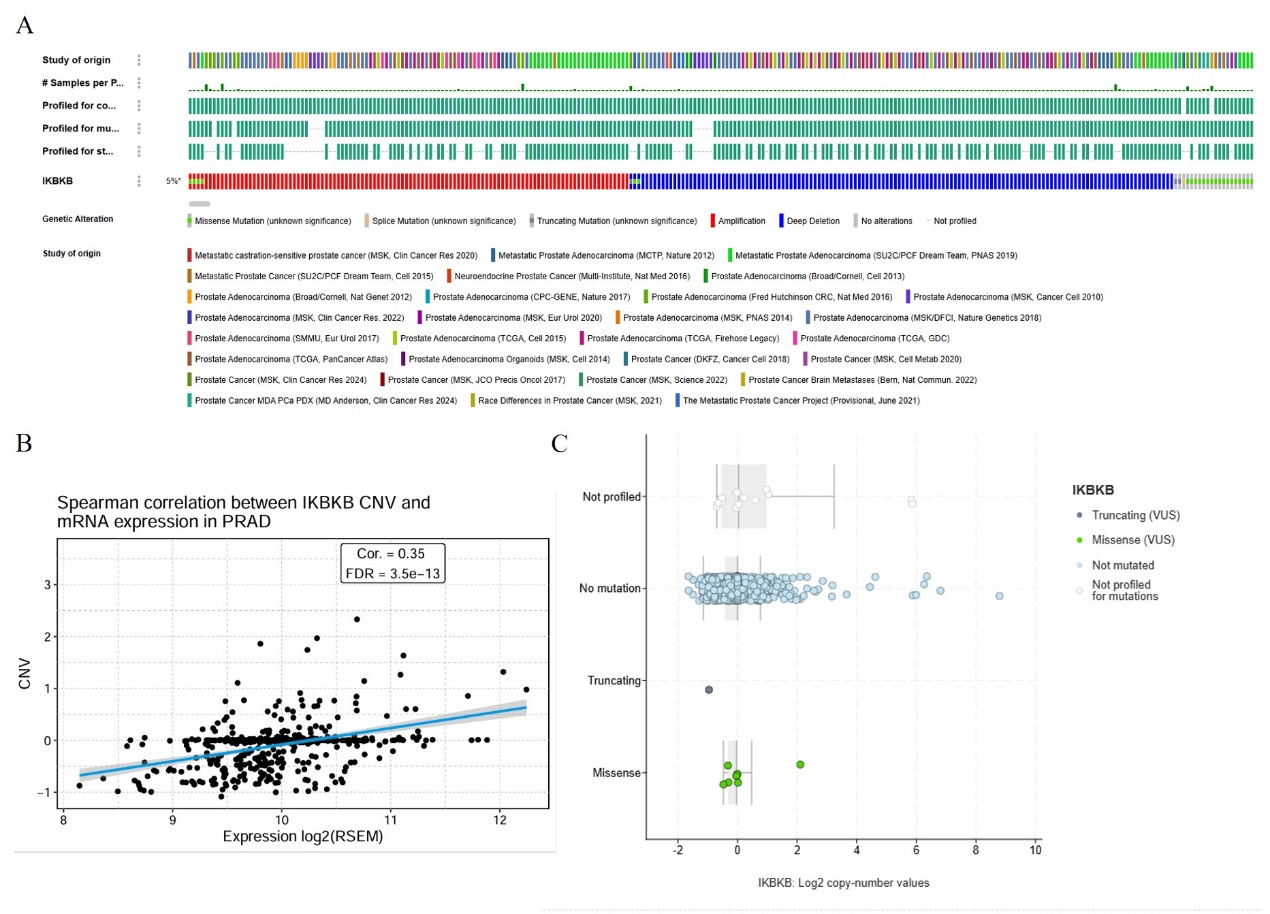


Figure S1. The relation between IKBKB expression and CNV. (A) Mutation frequency of IKBKB in PRAD. (B) Spearman correlation between IKBKB CNV and mRNA expression in PRAD. (C) The details for IKBKB copy number values and its CNV types.


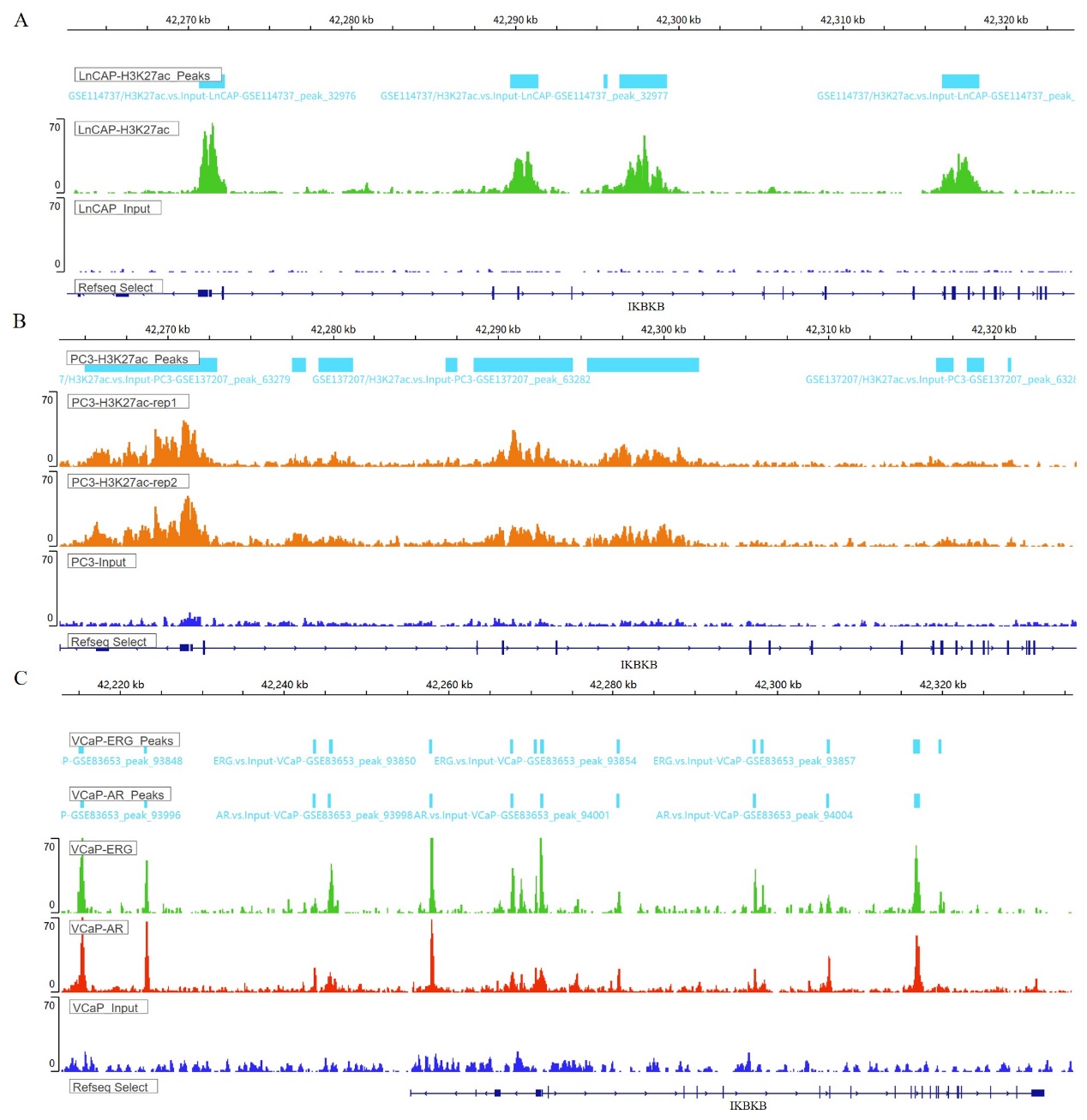


Figure S2. The Chip-seq data for IKBKB in PRAD. (A) LnCAP-H3K27ac (GSE114737). (B) PC3-H3K27ac (GSE137207). (C) VCap (GSE83653).


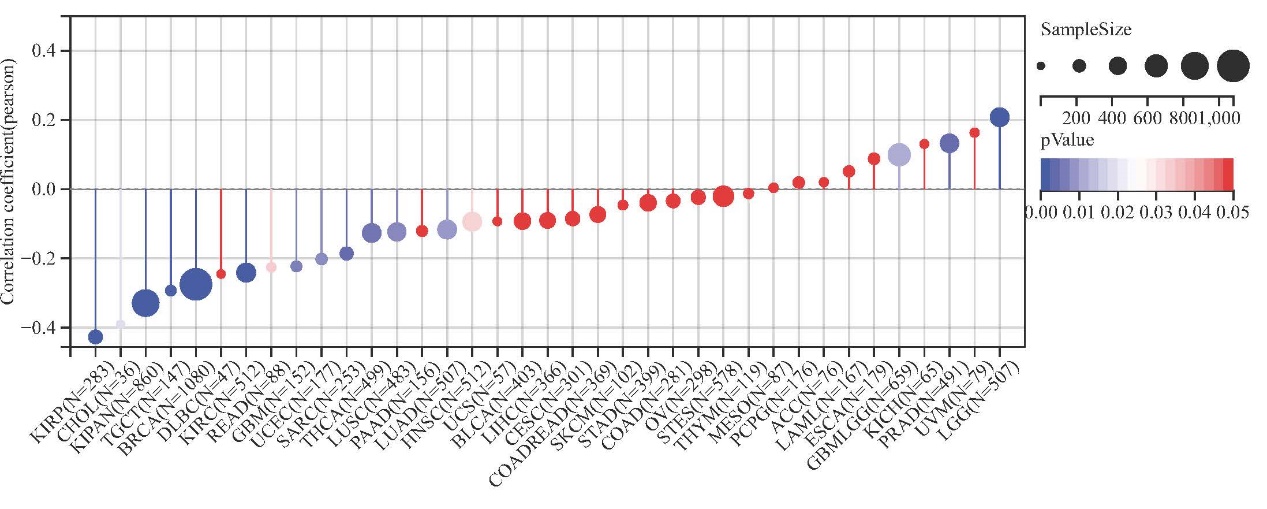


Figure S3. EREG.EXPss values for IKBKB in all types of cancer.


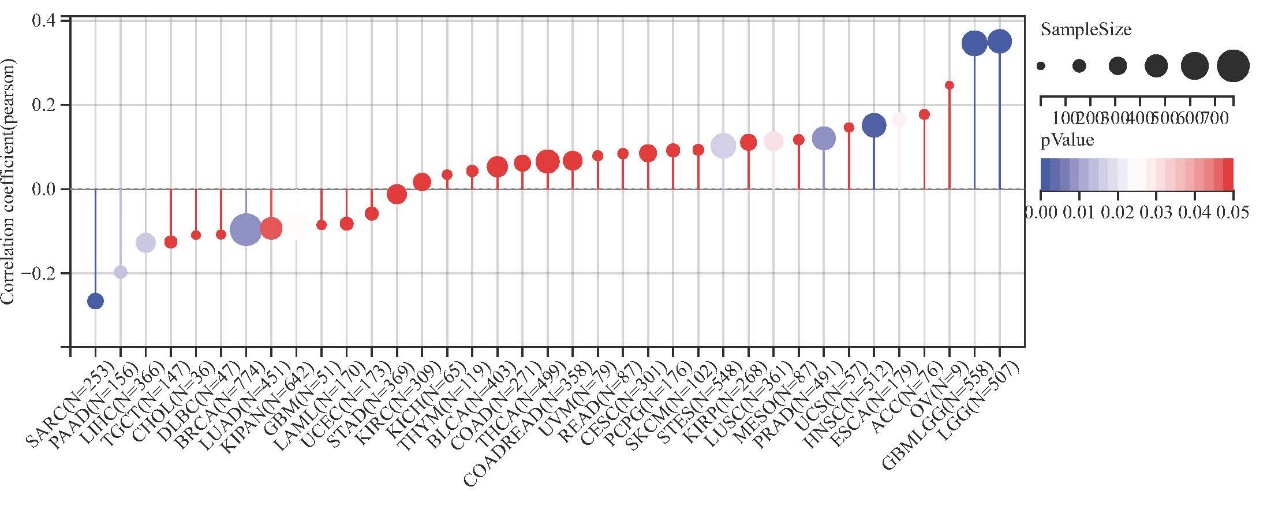


Figure S4. ENHss values for IKBKB in all types of cancer.


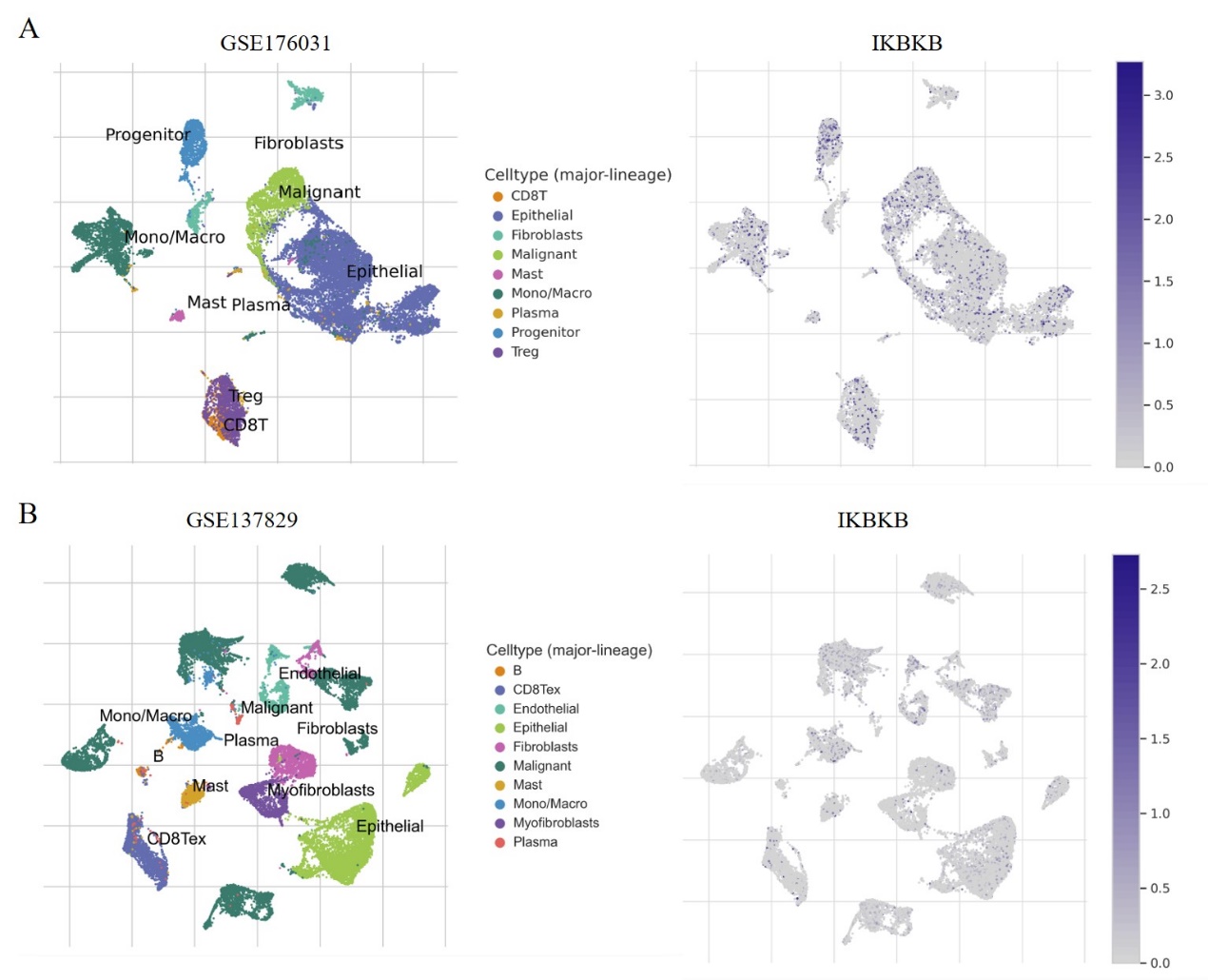


Figure S5. IKBKB expression in single cell data. (A) IKBKB expression in GSE176031. (B) IKBKB expression in GSE136829.


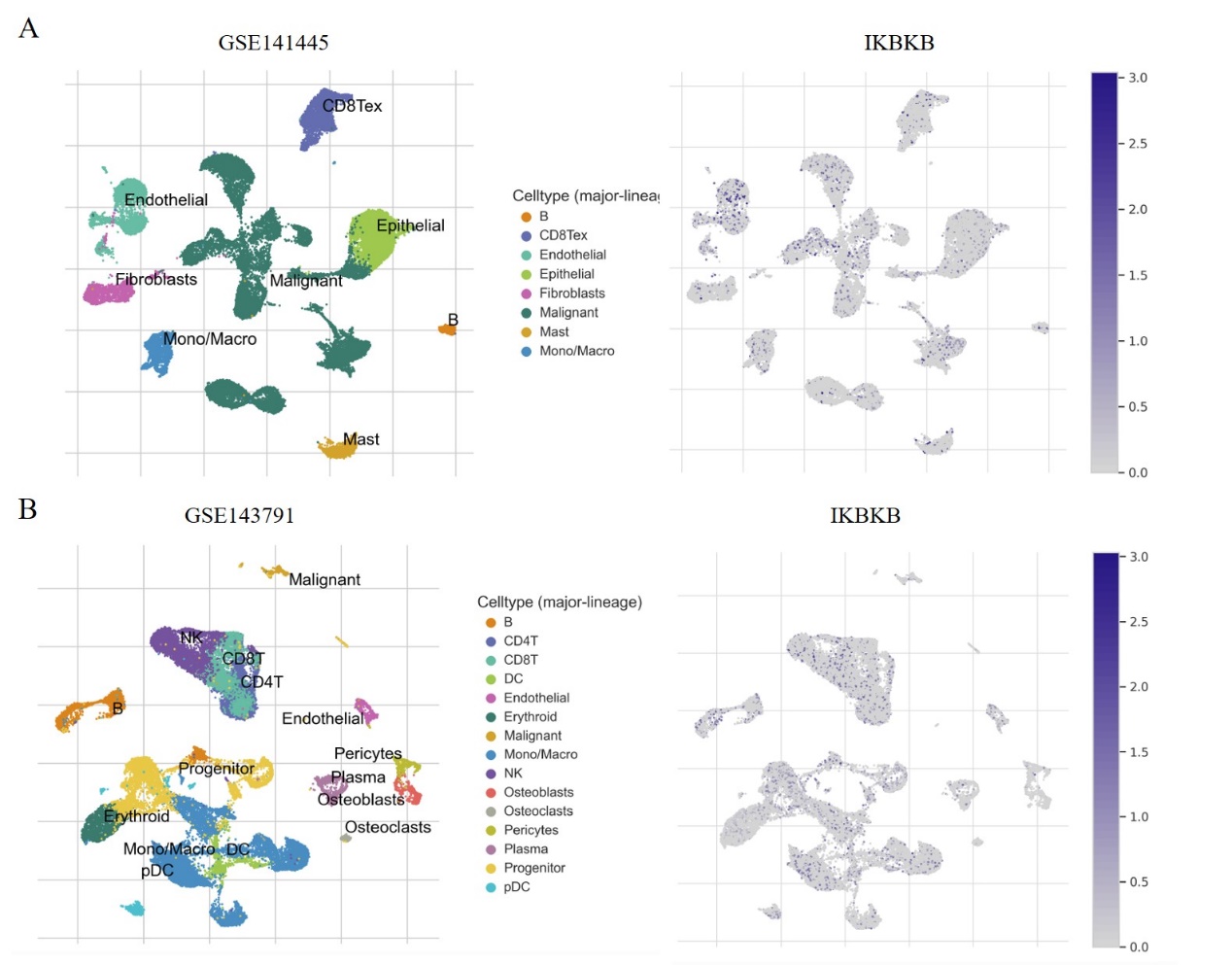


Figure S6. IKBKB expression in two prostate cancer single cell data. (A) IKBKB expression in GSE141445. (B) IKBKB expression in GSE143791.


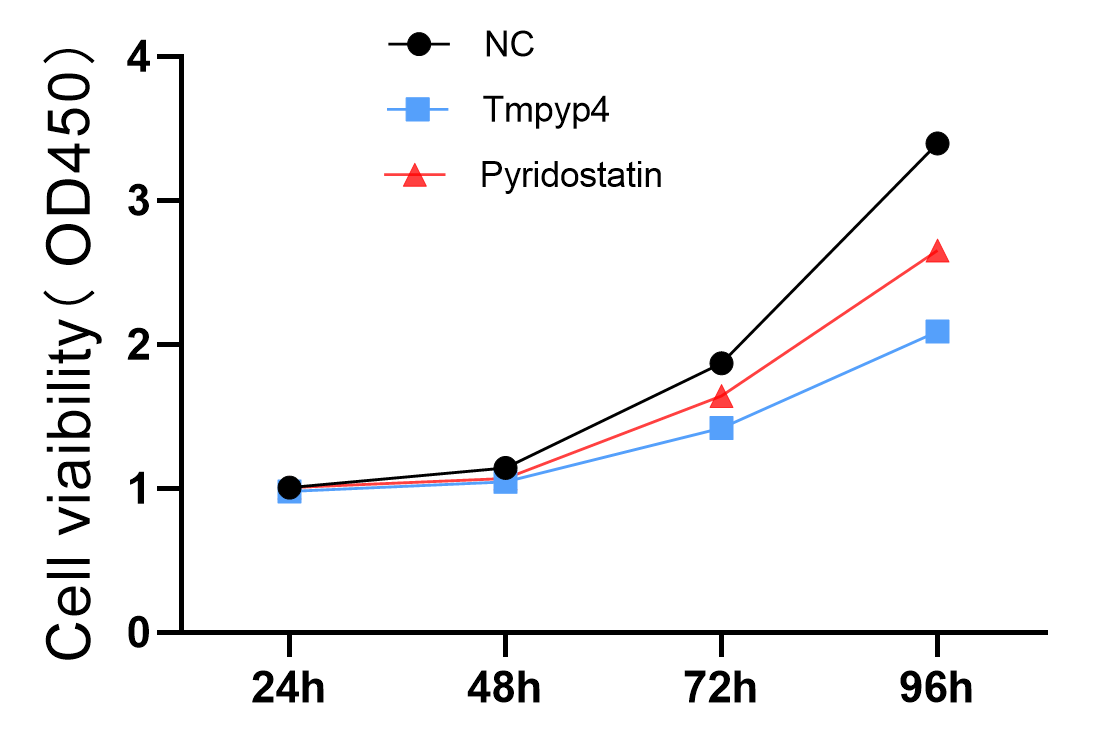


Figure S7. The OD450 values for C4-2 cells with 20 µM drug treatments. NC represents negative control.


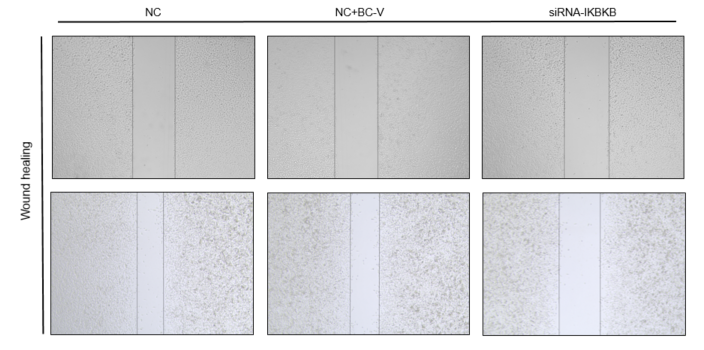


Figure S8. IKBKB plays an activating role in prostate cancer progress. BC-V represents empty vector.
